# Key Factors of Adherence in Cardiological Follow-Up of Adults with Congenital Heart Disease

**DOI:** 10.3390/jcdd12020039

**Published:** 2025-01-24

**Authors:** Anna-Lena Ehmann, Emily Schütte, Janina Semmler, Felix Berger, Ulrike M. M. Bauer, Katharina Schmitt, Constanze Pfitzer, Paul C. Helm

**Affiliations:** 1 Department of Congenital Heart Disease—Pediatric Cardiology, Deutsches Herzzentrum der Charité, Augustenburger Platz 1, 13353 Berlin, Germany; anna-lena.ehmann@charite.de (A.-L.E.); emily.schuette@charite.de (E.S.);; 2 Charité—Universitätsmedizin Berlin, Corporate Member of Freie Universität Berlin and Humboldt-Universität zu Berlin, Charitéplatz 1, 10117 Berlin, Germany; 3 National Register for Congenital Heart Defects, Augustenburger Platz 1, 13353 Berlin, Germany; 4 Department of Obstetrics, Charité—Universitätsmedizin Berlin, Corporate Member of Freie Universität Berlin and Humboldt-Universität zu Berlin, Augustenburger Platz 1, 13353 Berlin, Germany; 5 Competence Network for Congenital Heart Defects, Augustenburger Platz 1, 13353 Berlin, Germany; 6 Department of Psycho-Cardiology, Deutsches Herzzentrum der Charité, Augustenburger Platz 1, 13353 Berlin, Germany

**Keywords:** congenital heart defect, adherence, illness perception, follow-up, cardiac care

## Abstract

Approximately 50% of adults with congenital heart defects (ACHD) lack specialised CHD care, increasing the risk of preventable complications and mortality. While there is evidence that psychological factors significantly influence adherence, predictors of attending cardiological routine examinations in ACHD remain understudied. This is the first German study to examine psychological and sociodemographic predictors of adherence in ACHD using the Common-Sense Model of Self-Regulation as a framework. A total of N = 1136 participants from the National Register for Congenital Heart Defects were analysed. Sociodemographic and psychological factors (illness perception, illness identity, emotion regulation and psychological distress) were recorded as predictors of the subjective importance of regular cardiological check-ups and the actual utilisation frequency. The results indicate that of the sociodemographic factors, only age is relevant for the subjective importance, while net income influences the actual utilisation of cardiological examinations. In contrast, several psychological aspects of illness perception, such as perceived treatment benefit, and illness identity play a role for both adherence measures, as do depressive symptoms for the frequency of examinations. Our results highlight the importance of addressing psychological factors and providing clear information about the benefits of cardiological care to improve adherence in ACHD and thereby reduce secondary diseases.

## 1. Introduction

Adherence describes compliance with the treatment goals agreed upon by the patient and doctor in the context of medical treatment [1]. Studies indicate that around 50% of adults with congenital heart defects (ACHD) do not take up regular cardiological treatment, which is associated with increased morbidity and mortality, particularly in patients with high-risk secondary diseases [2,3,4]. For this reason, the topic of adherence should be given more consideration in research in order to further improve the care of ACHD. Over 90% of all patients with a congenital heart defect (CHD) now reach adulthood due to medical advances [5]. Regardless of the complexity of the CHD, the disease requires lifelong medical monitoring in most cases [2,6,7].

Previous research suggests that the utilisation of medical care services in ACHD is dependent not only on the complexity of the CHD but also on psychological aspects [8]. In the literature, psychological factors such as disease perception, illness identity and psychological distress are discussed as relevant variables in connection with different measures of adherence, such as medication adherence, primary care visits or adherence to therapeutic regimens in general [9,10,11,12,13,14]. However, little is known about the relationships between psychological factors, coping processes with illness and adherence in ACHD, specifically in relation to cardiological routine and control examinations.

The Common-Sense Model of Self-Regulation (CSM) provides a useful framework for understanding coping processes related to adherence in illness management [15]. In addition to sociodemographic and dispositional factors, the CSM includes psychological variables such as illness perception, illness identity, mental well-being and emotion-focused coping strategies and therefore provides a theoretical framework for identifying relevant correlates of adherence. Reciprocal mechanisms of action between the variables are assumed. The model was originally developed for acute illnesses but has since been transferred to chronically ill patients [10]. A fundamental hypothesis of the model with regard to adherence is that the individual’s perception of the disease is significantly linked to health-related actions, such as consulting a doctor. Specifically, according to the theory, non-adherence is more likely to occur if no symptoms or limitations are perceived, i.e., the patient sees no need to seek medical treatment [15].

Several studies have already investigated the psychological variables included in the CSM in the context of adherence and report indications of relevant correlations; in particular, illness perception has been shown to play a significant role in healthcare utilisation across different disease patterns [10,12,13,16,17,18]. Among patients with CHDs, individuals who perceive a strong impact of the disease on daily life, regardless of CHD severity or physical functioning, appear more likely to use outpatient (cardiological) treatment options [8]. Patients with a greater sense of personal control over their own illness seem to exhibit a more preventive style of action and tend towards adherent behaviour [10,14].

The construct of illness identity, which provides information on the extent to which a chronic illness is integrated into one’s identity and affects self-perception, also appears to be associated with adherence [19,20]. An adaptive illness identity, characterised by acceptance, is correlated with better individual disease management [21], while a rejecting illness identity, on the other hand, has been associated with problematic self-management and lower adherence [20,22]. In contrast, there have also been studies that positively linked a rather poorer disease identity with adherence [23].

In the context of emotion-focused coping strategies, the literature to date provides evidence that a more adaptive approach to emotions (e.g., acceptance) is associated with improved adherence and better disease-related self-management [10,11]. Furthermore, depression, but also anxiety and childhood trauma, in the context of cardiovascular and other physical diseases such as diabetes, represents a risk for poor adherence [9,24,25,26,27].

Overall, the studies mentioned above provide scientific evidence that the psychological factors included in the CSM play a significant role in the context of adherence. Previous studies have only investigated adherence in ACHD in relation to diet, medication intake or cardiac rehabilitation, for example [9,28,29]. However, to date, there has been no study investigating potential psychological predictors of adherence in the context of routine and control examinations in ACHD. This study is the first to simultaneously explore the constructs outlined in the CSM and evaluate whether psychological factors and sociodemographic variables significantly influence the utilisation of routine cardiological examinations and check-ups in ACHD. The findings of our study therefore contribute to a more comprehensive understanding of the relationships between relevant variables in the context of coping with illness and adherence. In the long term, the results should provide evidence-based approaches for improving adherence and reducing secondary diseases in this patient group.

## 2. Materials and Methods

### 2.1. Study Design

A total of 1136 ACHD from the National Register for Congenital Heart Defects (NRCHD) were included in this online-based cross-sectional study. The data were collected as part of an online survey that examined adherence, coping strategies and mental health in ACHD patients. Participants were notified via email and completed self-reported questionnaires regarding sociodemographic information, the utilisation of cardiological examinations, illness perception and identity, mental health and emotion regulation. Additionally, medical information from the NRCHD database was incorporated into the statistical analyses, with cardiac diagnoses classified according to the International Pediatric and Congenital Cardiac Code (IPCCC) [30]. Data acquisition was carried out during the first quarter of 2024.

### 2.2. National Register for Congenital Heart Defects

As of December 2024, the NRCHD stands as Europe’s most comprehensive database for CHDs, encompassing medical data from approximately 60,000 CHD patients. Accordingly, the NRCHD is a solid basis for clinical research in the field of CHDs [31]. Participation in the NRCHD is voluntary and requires patients to grant general consent. This consent explicitly allows the NRCHD to collect and store medical records from treating physicians for use in current and future studies, with patients retaining the option to withdraw their consent at any time.

### 2.3. Measures

#### 2.3.1. Illness Perception

Illness perception was recorded using the Brief Illness Perception Questionnaire (BIPQ) [32]. This questionnaire consists of nine items that capture cognitive and emotional representations of the illness. It includes experience of control, treatment benefit, perceived symptoms and impairment of the disease, (emotional) involvement, worries about the disease and understanding of the disease. We omitted the items on the estimated duration and suspected causes of the disease due to the fact that CHDs are a chronic, congenital disease. Each item is answered on a scale of 0–10, with 0 being the weakest expression. A mean value is calculated for each item.

#### 2.3.2. Illness Identity

The Illness Identity Questionnaire (IIQ) [20] was used to measure illness identity. The questionnaire measures the extent to which respondents are able to integrate their illness into their own identity and contains four different dimensions of illness identity: while *engulfment* and *rejection* imply insufficient integration of the illness into the self, *acceptance* and *enrichment* are seen as adaptive forms of illness identity. Each of the four dimensions is measured by several items, which are rated on a scale from 1 (lowest expression) to 5 (highest expression). Mean values are calculated for each dimension.

#### 2.3.3. Mental Health

##### HADS

The Hospital Anxiety and Depression Scale (HADS) [33] is widely used in somatic and psychosomatic contexts. The scale includes 14 items, divided into two subscales: 7 items assess depression (HADS-D) and 7 assess anxiety (HADS-A). Each item is rated on a four-point scale, with total scores ranging from 0 to 21. The HADS is designed to focus on non-physical symptoms in order to reduce potential confounding effects from physical illness [34]. Symptom severity is classified as mild (8–10 points), moderate (11–14 points) or severe (15–21 points).

##### PHQ-9

Comprising nine items, the Patient Health Questionnaire-9 (PHQ-9) [35] aims at evaluating the severity of depressive symptoms. These items inquire about experiences within the past two weeks and are scored on a four-point scale. Based on the total score, which ranges from 0 to 27, the severity of depressive symptoms is classified into four categories: mild (5–9 points), moderate (10–14 points), moderately severe (15–19 points) or severe (20–27 points).

##### GAD-7

The severity of anxiety symptoms was assessed by the Generalized Anxiety Disorder-7 (GAD-7) [36] consisting of seven items. Participants rate the extent to which anxiety symptoms interfere with their daily functioning on a four-point scale. The total GAD-7 score ranges from 0 to 21, with higher scores indicating greater severity of anxiety symptoms. Severity is classified using the following cut-off values: mild (5 points), moderate (10 points) and severe (15 points).

#### 2.3.4. Emotion Regulation

The use of eight emotion regulation strategies was measured by the Heidelberg Form for Emotion Regulation Strategies (H-FERST) [37], which includes 28 items evaluating strategies such as reappraisal, problem-solving, acceptance, social sharing, rumination, avoidance, expressive suppression and suppression of experience. Participants rate the items on a 5-point scale, ranging from 1 = never to 5 = always. The reliability of all subscales exceeds α = 0.80.

#### 2.3.5. Adherence

Adherence was measured in two different ways: Firstly, the participants were asked how important they consider regular cardiological check-ups to be on a scale from 1 = not at all important to 6 = very important. Secondly, the participants were asked at what intervals they usually go to the cardiologist. This information was coded on an ordinal scale of 1–8 as follows: intervals of >5 years or irregular intervals = 1, every 5 years = 2, every 4 years = 3, every 3 years = 4, every 2 years = 5, every 1.5 years = 6, once a year = 7, several times a year = 8.

#### 2.3.6. Sociodemographic Factors

We included age, sex, net income based on a median split (EUR ≤ 3000 vs. EUR > 3000), residence size, number of years at school and relationship status (firm relationship yes/no) as sociodemographic factors in the analyses.

### 2.4. Statistical Analyses

The data were analysed using SPSS (Version 29.0). We calculated two different ordinal regression models to evaluate potential predictors for (1) the subjective importance of regular cardiological check-ups and (2) the actual frequency of cardiological check-ups. Aspects of illness perception, illness identity, symptoms of depression and anxiety, emotion regulation strategies, sociodemographic variables and CHD severity were included as independent variables in both regression models. Bonferroni correction was performed based on the calculation of two regression models. The significance level for independent variables was thus set at *p* < 0.025. Chi-square tests were performed for group comparisons.

### 2.5. Ethical Statement

The Charité issued a favourable ethics vote for this study (EA4/178/22). Participants gave their written informed consent to participate in the study.

## 3. Results

### 3.1. Study Cohort

A total of N = 1468 patients took part in the survey. For the analyses, patients with insufficient medical information on the severity of their CHD (N = 223) and participants who did not provide any information on their net income (N = 127) were excluded. The sample considered for the analyses consisted of N = 1136 patients aged 18 to 85 (M_age_ = 36.73 years, SD_age_ = 14.03). Of these, 678 (59.7%) participants were women and 458 (40.3%) were men. In all, 142 (12.5%) participants had a simple CHD, 618 (54.4%) a moderate CHD and 376 (33.1%) a complex CHD. Chi-square tests showed a significant gender difference in the distribution of CHD severities (*p* < 0.05). Table 1 displays the sociodemographic characteristics of the total sample and each CHD severity level.

### 3.2. Subjective Importance of Regular Check-Ups

The importance of regular cardiological check-ups was rated as high overall (M = 5.32, SD = 1.16, range: 1–6). The ordinal regression with the rating of the subjective importance of regular check-ups as a dependent variable revealed only age (*p* < 0.025, odds ratio (OR): 1.01 [CI: 1.00–1.03]) as a significant sociodemographic predictor. Among the psychological factors, aspects of illness perception in particular, such as subjective impairment (*p* < 0.001, OR: 1.22 [CI: 1.10–1.35]), perceived control (*p* < 0.001, OR: 0.89 [CI: 0.85–0.93]), treatment benefit (*p* < 0.001, OR: 1.30 [CI: 1.23–1.37]) and worries about the disease (*p* < 0.001, OR: 1.26 [CI: 1.16–1.36]), were significant.

Of the dimensions of illness identity, only *enrichment* (*p* < 0.025, OR: 1.23 [CI: 1.05–1.44]) was significant. There was no significant effect for emotion regulation strategies or for symptoms of depression and anxiety (*p* > 0.025 for all). A representation of the significant odds ratios can be found in Figure S1 in the Supplementary Material.

**Figure 1:**
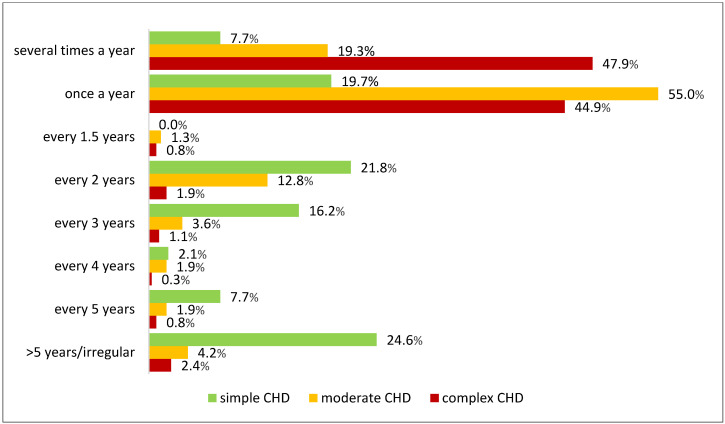
Percentage frequency of cardiological check-ups according to CHD severity.

The ordinal regression model showed no significant gender difference in the importance of check-ups (*p* = 0.836). In contrast, CHD severity had a significant effect on the rating of the subjective importance (*p* < 0.001), which can be seen in Figure 1. Table S1 shows the results of the ordinal regression model with the subjective importance of regular cardiological check-ups as a dependent variable and can be viewed in the Supplementary Material.

### 3.3. Frequency of Cardiological Check-Ups

**Figure 2:**
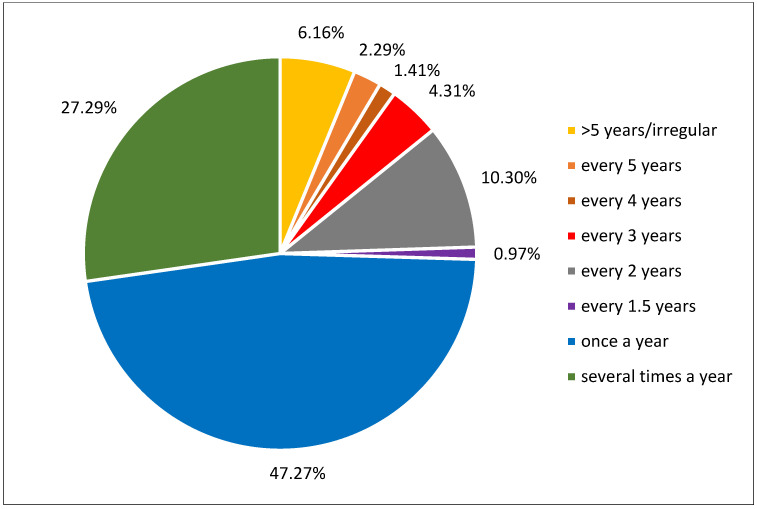
Percentage frequencies of utilisation of cardiological check-ups for the total sample.

Figure 2 displays the percentage frequencies of the utilisation of cardiological check-ups for the total sample in detail.

Considering sociodemographic factors, the ordinal regression with the frequency of cardiological check-ups as a dependent variable revealed only net income as a significant predictor (*p* < 0.025, OR: 1.40 [CI: 1.08–1.81]).

Among the aspects of illness perception, subjective impairment (*p* < 0.001, OR: 1.23 [CI: 1.13–1.33]), treatment benefit (*p* < 0.001; OR: 1.12 [CI: 1.07–1.18) and worries about the CHD (*p* < 0.025, OR: 1.09 [CI: 1.02–1.17]) were significant.

Moreover, depressive symptoms (*p* < 0.025, OR: 0.94 [CI: 0.90–0.98]), measured by the PHQ-9, and feeling engulfed by the disease (*p* < 0.025, OR: 1.58 [CI: 1.18–2.12]) showed a significant influence. There was no significant effect for emotion regulation strategies (*p* > 0.025 for all). A representation of the significant odds ratios is displayed in Figure S2 in the Supplementary Material.

**Figure 3:**
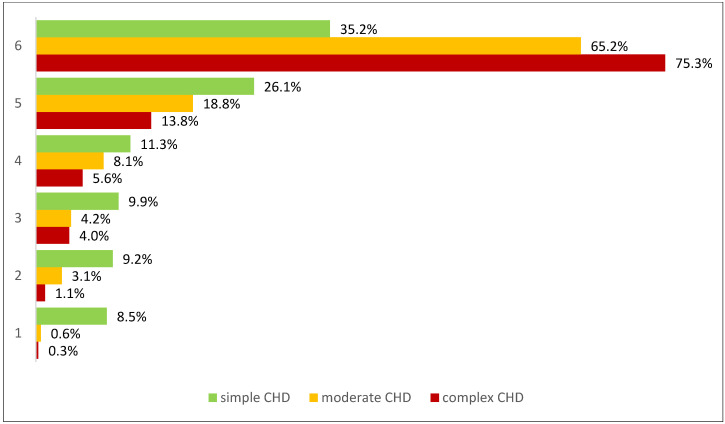
Assessment of the subjective importance of regular cardiological check-ups (from 1= not important at all to 6 = very important) as a percentage depending on CHD severity.

While there was no significant effect of gender on the intervals between check-ups (*p* = 0.313), a significant difference was found between CHD severities (*p* < 0.001) in relation to the intervals between the control examinations, which is illustrated in Figure 3. For detailed results of the ordinal regression model with intervals between cardiological check-ups as a dependent variable, Table S2 can be found in the Supplementary Material.

## 4. Discussion

This study is the first to analyse psychological and sociodemographic predictors of adherence in ACHD, based on the CSM. As measures of adherence, we examined the subjective importance of regular cardiological check-ups on the one hand and the actual frequency of these check-ups on the other. Our results show that overall, the majority of respondents attend regular check-ups, and the importance of these is rated as high on average.

CHD severity showed an influence on the adherence measures we recorded: a higher severity increases the probability of going to cardiological check-ups more often and rating them as subjectively more important, which can be comprehensibly explained by the severity of the disease and associated comorbidities. This result is in line with a previous study on healthcare use in ACHD [8].

In contrast to psychological factors, sociodemographic variables appear to have a limited influence on the adherence measures we recorded. Only a lower net income seems to increase the probability of a more frequent use of cardiological examinations. This result contrasts with a number of studies that found a lower income to be associated with poorer adherence across different disease patterns [38,39,40,41,42]. One explanation for our contrary finding might be that patients with a more severe CHD also tend to have a lower net income, for example, due to part-time work or barriers in achieving a higher level of education. Consequently, there may be more patients with more severe CHDs among lower earners, and it is not the lower income per se that leads to more frequent utilisation of examinations but the associated severity of illness. For the assessment of subjective importance, only a higher age was associated with a higher probability of a subjectively higher rating of importance. Sex, size of residence and number of school years, on the other hand, proved to be irrelevant for both the subjective assessment and for the actual utilisation of routine examinations. Other studies on adherence also identified age [23,43] as a relevant factor but with opposing effects, along with lower education level [43] and male gender [27] being associated with lower adherence.

With regard to the psychological factors, our results provide evidence that aspects of illness perception, illness identity and symptoms of depression are significantly associated with adherence.

For both the subjective importance of regular check-ups and the actual frequency of utilisation of cardiological examinations, subjective impairment and perceived treatment benefit were significant predictors. A stronger perceived impairment and a higher subjective treatment benefit increase the probability of a more frequent utilisation of cardiological check-ups and a higher assessment of their importance. While the perceived impairment appears to be less influenceable, education in the context of medical treatment should therefore be accorded great relevance. Whenever possible, practitioners should take the time to discuss the goals and background of the treatment with the patient. A study on healthcare utilisation in ACHD found that patients were more likely to use healthcare services if they perceived their illness as having a significant impact on their lives and believed that their CHD could be managed either through treatment or on their own [8]; this corresponds with our results.

Furthermore, the more controllable patients perceive their illness to be, the less important they consider regular examinations. A sense of helplessness may therefore enhance patients’ willingness to actively manage their health. Interestingly, an opposite effect was found for other diseases such as diabetes or hypertension [10,14]. Possibly, other factors such as CHD severity or trust in the treating physician play a role in the context of perceived control in our group of patients.

In terms of the subjective importance of check-ups, psychological stress such as depressive or anxious symptoms does not appear to play a significant role. However, the situation is different with regard to the actual check-up intervals: depressive symptoms, measured with the PHQ-9, showed a significant influence on the frequency of utilisation of routine examinations. This result indicates that more severe depressive symptoms are associated with lower utilisation of cardiological examinations. An obvious explanation for this might be typical symptoms such as listlessness or self-neglect. Patients with depressive symptoms should therefore be given targeted support in making use of medical examinations. The need for regular check-ups should be particularly emphasised for this vulnerable patient group. This result is also consistent with multiple studies that have linked higher psychological stress with lower adherence [24,25,26,27]. Increased anxiety symptoms did not show a significant effect on our measures of adherence but have primarily been associated with medication adherence in the existing literature [9]. It should be noted that different screening tools tend to produce different results in the recording of psychological symptoms [44], which is also evident in the results of our regression models considering depressive symptoms.

Specific worries about the disease also appear to have an influence, with an opposite effect compared to depression: patients with increased worries about their CHD tend to make more frequent use of check-ups and rate their importance as higher. The fact that increased symptoms of depression reduce the likelihood of more frequent cardiological examinations, while increased illness-related worries raise the likelihood of frequent check-ups, suggests the need for a more in-depth investigation into the exact factors involved in the context of depressive illnesses. It could be assumed that in the context of depressive symptoms, it is not so much the cognitive and emotional symptoms but rather behavioural components that are decisive for lower adherence. In addition, it could be surmised that it is important to differentiate whether worries or ruminations in the context of depression are more irrational, which seems to lead to lower adherence, or whether they are justified illness-related worries that reflect reality and, in contrast, lead to higher adherence.

This hypothesis is supported by the fact that the illness identity dimension *engulfment*, which implies a pronounced subjective dominance of the CHD in relation to one’s own life, thoughts, feelings and activities, seems to go hand in hand with a higher probability of attending check-ups more frequently. A form of illness identity that is rather maladaptive for psychological well-being thus appears to have a positive effect on adherence in ACHD. Our results provide evidence that illness identity also plays a partial role in the assessment of subjective importance: it was shown that patients who see their illness as *enriching* are more likely to rate the subjective importance of regular examinations more highly. Just like our results in the area of illness identity, previous studies have produced contradictory results in this context. While some studies have found that a more adaptive illness identity such as higher acceptance or lower rejection is associated with better adherence [21,22], there are also studies that have found the opposite effect [23]. This calls for the dimensions of illness identity in the context of adherence to not be categorised across the board as adaptive or maladaptive.

Contrary to the assumption based on previous studies [10,11], our results provide no evidence that emotion regulation strategies have an influence on the adherence measures we recorded.

Some strengths and limitations of our study should be emphasised at this point. The novelty of this study lies in addressing the gap in adherence research among ACHD in Germany, which stands out as a notable strength. Additionally, we successfully recruited a large cohort of ACHD in Germany, encompassing varying levels of disease severity. Despite the higher proportion of female participants, the sample can be deemed sufficiently representative [31]. Meta-analyses criticise the inconsistent operationalisation of adherence [45,46]. In fact, the operationalisation we have chosen is only one possible option. This should be taken into account when comparing the results with previous studies. The authors of the CSM assume dynamic mechanisms between the relevant constructs in the context of coping with illness [15]. Our cross-sectional survey of the constructs does not allow any conclusions to be drawn about causal mechanisms of action. Nevertheless, our results provide important information about the relationships between the individual variables in ACHD, which have not yet been fully investigated and can contribute to a more comprehensive understanding of coping with illness. There is a risk of bias with regard to the recording of adherence. For example, it could be that people who tend to be non-adherent deal less with disease-related topics overall and participate less frequently in corresponding studies and surveys. Moreover, these are subjective statements on adherence, which could be distorted, for example, by memories. With the dichotomous variable ‘firm relationship’, it should also be noted that the perception and interpretation of the relationship status can be subjective. The results of our ordinal regression model provided evidence of a violation of the proportionality assumption. Although the proportional odds assumption may not be fully satisfied in our model, it still provides a robust estimate of the overall effect of psychological and sociodemographic factors on the frequency of medical check-ups and the subjective evaluation of their importance. The violation of the assumption suggests that the influence of these factors may differ across categories of the dependent variables, which could be investigated in future analyses with more flexible models [47].

## 5. Conclusions

In summary, our study shows that among sociodemographic factors, net income plays a significant role in the utilisation of cardiological check-ups, whereas age is primarily associated with the subjective assessment of their importance. In addition, multiple psychological factors such as illness perception, illness identity and depressive symptoms appear to influence the adherence measures recorded, along with CHD severity. Our results further indicate that psychological stress in the sense of increased worries or a greater sense of being taken over by the CHD is not necessarily associated with lower adherence. There seems to be a difference between irrational worries or rumination in the context of depression and specific disease-related worries, which appear to have opposite effects on adherence. A negative effect of behavioural depressive symptoms such as listlessness on adherence can also be assumed. Finally, our results indicate that providing information about the benefits of cardiological treatment can be an important starting point for the use of regular preventive examinations.

In the long term, the results of our study provide a foundation for optimising adherence in ACHD, potentially reducing the prevalence of secondary diseases recorded by Diller et al. [3]. Studies in the context of chronic diseases have already shown that the modification of psychological predictors, such as coping strategies or quality of life, through targeted interventions can actually influence the use of healthcare and treatment services [48,49,50]. Interventions targeting illness perception also showed positive effects on quality of life, psychopathology and adherence [51,52,53].

Our results suggest that psychological aspects such as illness perception, illness identity and depressive symptoms should be given greater consideration in patient care. In addition, this study emphasises the importance of clear and understandable information about the benefits of cardiological treatments and regular check-ups. Patients should be actively informed about the benefits of preventive measures in order to encourage their utilisation. Psychotherapeutic interventions should aim to reduce depressive behavioural symptoms such as listlessness, as these can have a negative impact on adherence.

## Figures and Tables

**Table 1:** Demographic characteristics of the total sample and for each of the CHD severity levels.

Demographic Characteristic	Total Sample (N = 1136)	Simple CHD (N = 142)	Moderate CHD (N = 618)	Complex CHD (N = 376)
**Age in years, M (SD)**	36.7 (14.0)	34.2 (14.9)	38.5 (14.4)	34.8 (12.6)
**Sex, n (%)**				
Male	458 (40.3)	44 (31.0)	264 (42.7)	150 (39.9)
Female	678 (59.7)	98 (69.0)	354 (57.3)	226 (60.1)
**Relationship status, n (%)**				
Single	404 (35.6)	50 (35.2)	203 (32.8)	151 (40.2)
In a relationship/married	732 (64.4)	92 (64.8)	415 (67.2)	225 (59.8)
**Number of school years, M (SD)**	11.37 (1.6)	11.42 (1.3)	11.38 (1.5)	11.35 (1.7)
**Education, n (%)**				
Without degree	29 (2.6)	2 (1.4)	15 (2.4)	12 (3.2)
Pupil	14 (1.2)	0 (0.0)	11 (1.8)	3 (0.8)
Elementary school	35 (3.1)	0 (0.0)	19 (3.1)	16 (4.3)
Secondary school	105 (9.2)	15 (10.6)	54 (8.7)	36 (9.6)
Completed apprenticeship	250 (22.0)	26 (18.3)	142 (23.0)	82 (21.8)
Advanced technical college	117 (10.3)	21 (14.8)	59 (9.5)	37 (9.8)
High school diploma	175 (15.4)	23 (16.2)	101 (16.3)	51 (13.6)
University	396 (34.9)	54 (38.0)	210 (34.0)	132 (35.1)
Other	15 (1.3)	1 (0.7)	7 (1.1)	7 (1.9)
**Employment, n (%)**				
School	24 (2.1)	2 (1.4)	13 (2.1)	9 (2.4)
Trainee	61 (5.4)	13 (9.2)	23 (3.7)	25 (6.6)
University	129 (11.4)	22 (15.5)	71 (11.5)	36 (9.6)
Part-time job	262 (23.1)	33 (23.2)	132 (21.4)	97 (25.8)
Full-time job	496 (43.7)	60 (42.3)	286 (46.3)	150 (39.9)
Seeking a job	16 (1.4)	2 (1.4)	8 (1.3)	6 (1.6)
Independent	57 (5.0)	7 (4.9)	32 (5.2)	18 (4.8)
Retired	135 (11.9)	10 (7.0)	73 (11.8)	52 (13.8)
Other	55 (4.8)	5 (3.5)	29 (4.7)	21 (5.6)
**Net income, n (%)**				
EUR ≤ 3.000	624 (54.9)	78 (54.9)	323 (52.3)	223 (59.3)
EUR > 3.000	512 (45.1)	64 (45.1)	295 (47.7)	153 (40.7)
**Size of residence, n (%)**				
≤5.000	227 (20.0)	28 (19.7)	111 (18.0)	88 (23.4)
5.001–20.000	307 (27.0)	44 (31.0)	169 (27.3)	94 (25.0)
20.001–100.000	247 (21.7)	30 (21.1)	140 (22.7)	77 (20.5)
100.001–500.000	184 (16.2)	23 (16.2)	101 (16.3)	60 (16.0)
>500.000	171 (15.1)	17 (12.0)	97 (15.7)	57 (15.2)

## Data Availability

Data cannot be shared for data protection reasons.

## Supplementary Materials

The following supporting information can be downloaded at: https://www.mdpi.com/article/10.3390/jcdd12020039/s1, Table S1: Results of ordinal regression with the subjective importance of regular cardiological check-ups as the dependent variable and sociodemographic and psychological factors as well as CHD severity as independent variables; Figure S1: Odds ratios and 95%-CI intervals of significant independent variables in predicting the subjective importance of regular cardiological check-ups; Table S2: Results of ordinal regression with the frequency of cardiological check-up intervals as the dependent variable and sociodemographic and psychological factors as well as CHD severity as independent variables; Figure S2: Odds ratios and 95%-CI intervals of significant independent variables in predicting the frequency of cardiological check-up intervals.
