# Supplementary material for: Key Factors of Adherence in Cardiological Follow-Up of Adults with Congenital Heart Disease
Source: J Cardiovasc Dev Dis. 2025 Jan 24;12(2):39. doi: 10.3390/jcdd12020039 (PMC11856703; doi:10.3390/jcdd12020039)
Supplement: Supplementary file 1 [file jcdd-12-00039-s001.zip › jcdd-3425245-supplementary.pdf]

Table S1. *Results of ordinal regression with the subjective importance of regular cardiological check-ups as the dependent variable and sociodemographic and psychological factors as well as CHD severity as independent variables.*

		Odds ratio	**Standard Error**	**df**	***p***	**95% Confidence interval** lower	upper
Treshold	Subjective importance = 1	0,380	1,138	1	0,395	0,041	3,535
	Subjective importance = 2	1,552	1,121	1	0,695	0,172	13,978
	Subjective importance = 3	4,213	1,119	1	0,199	0,470	37,758
	Subjective importance = 4	10,518	1,119	1	0,036	1,172	94,345
	Subjective importance = 5	40,400	1,123	1	0,001	4,475	364,746
Location	Age	1,014	0,006	1	**0,020**	1,002	1,026
	School years	0,947	0,051	1	0,290	0,857	1,047
	Residence size	0,923	0,053	1	0,128	0,832	1,023
	IPQ_Impairment	1,219	0,052	1	**<.001**	1,101	1,349
	IPQ_Perceived control	0,886	0,023	1	**<.001**	0,847	0,927
	IPQ_Treatment Benefit	1,299	0,026	1	**<.001**	1,234	1,367
	IPQ_Perceived symptoms	0,907	0,050	1	0,051	0,822	1,000
	IPQ_Worries	1,257	0,041	1	**<.001**	1,161	1,361
	IPQ_Understanding	1,072	0,032	1	0,028	1,008	1,140
	IPQ_Emotional involvement	0,934	0,039	1	0,076	0,865	1,007
	HADS-A	0,973	0,030	1	0,363	0,916	1,033
	HADS-D	1,023	0,031	1	0,457	0,963	1,086
	PHQ-9	0,951	0,025	1	0,048	0,905	1,000
	GAD-7	0,997	0,032	1	0,929	0,937	1,062
	IIQ_Engulfment	1,349	0,181	1	0,098	0,946	1,923

IIQ_Rejection	1,017	0,102	1	0,870	0,833	1,241
IIQ_Acceptance	1,203	0,124	1	0,137	0,943	1,536
IIQ_Enrichment	1,228	0,080	1	**0,010**	1,050	1,436
HFERST_Rumination	1,008	0,089	1	0,927	0,847	1,200
HFERST_Reappraisal	1,020	0,099	1	0,839	0,840	1,239
HFERST_Acceptance	0,869	0,109	1	0,200	0,702	1,077
HFERST_Problemsolving	1,006	0,115	1	0,957	0,803	1,262
HFERST_Suppression of expression	0,954	0,108	1	0,663	0,771	1,180
HFERST_Suppression of experience	0,983	0,122	1	0,887	0,773	1,249
HFERST_Avoidance	0,962	0,089	1	0,663	0,808	1,145
HFERST_Social sharing	1,106	0,074	1	0,175	0,956	1,280
Male	1,032	0,151	1	0,836	0,768	1,387
Female	0[a]					
Firm relationship	1,185	0,157	1	0,279	0,871	1,612
Single	0[a]					
Net income <=3000€	1,214	0,150	1	0,197	0,904	1,630
Net income >3000€	0[a]					
CHD simple	0,412	0,226	1	**<.001**	0,265	0,642
CHD moderate	0,923	0,167	1	0,633	0,666	1,281
CHD complex	0[a]					

Link Function: Logit. a. This parameter is set to zero because it is redundant. p values in bold indicate significant effects of the predictors.

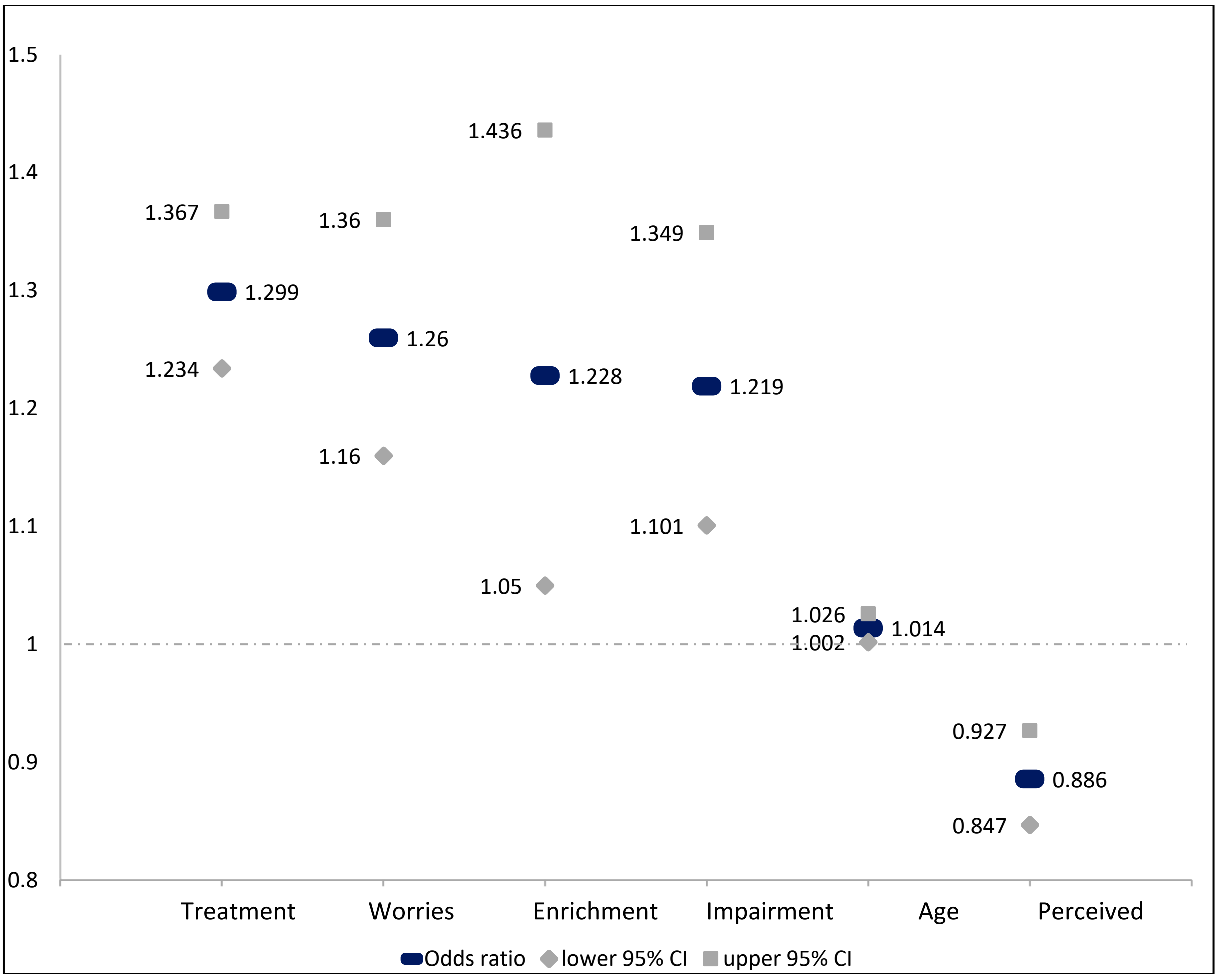

Figure S1. Odds ratios and 95%-CI intervals of significant independent variables in predicting the subjective importance of regular cardiological check-ups.

Table S2. *Results of ordinal regression with the frequency of cardiological check-up intervals as the dependent variable and sociodemographic and psychological factors as well as CHD severity as independent variables.*

						95 % Confidence interval	
		Odds ratio	**Standard Error**	**df**	***p***	lower	upper
Treshold	>5 years/ irregular	1,018	0,978	1	0,986	0,150	6,924
	Every 5 years	1,563	0,976	1	0,647	0,231	10,592
	Every 4 years	1,945	0,976	1	0,495	0,287	13,165
	Every 3 years	3,401	0,975	1	0,209	0,503	22,981
	Every 2 years	9,238	0,975	1	0,023	1,367	62,446

	Every 1.5 years	10,003	0,975	1	0,018	1,479	67,630
	Once a year	220,368	0,987	1	<.001	31,830	1525,659
Location	Age	0,999	0,005	1	0,842	0,989	1,009
	School years	1,004	0,043	1	0,925	0,924	1,091
	Residence size	0,963	0,047	1	0,423	0,879	1,055
	IPQ_Impairment	1,225	0,042	1	**<.001**	1,129	1,330
	IPQ_Perceived control	0,963	0,021	1	0,070	0,925	1,003
	IPQ_Treatment Benefit	1,123	0,024	1	**<.001**	1,072	1,177
	IPQ_Perceived symptoms	1,075	0,041	1	0,080	0,991	1,165
	IPQ_Worries	1,089	0,035	1	**0,013**	1,018	1,166
	IPQ_Understanding	0,962	0,029	1	0,174	0,909	1,017
	IPQ_Emotional involvement	1,012	0,034	1	0,727	0,947	1,081
	HADS-A	0,952	0,027	1	0,067	0,902	1,004
	HADS-D	1,055	0,027	1	0,049	1,000	1,114
	PHQ-9	0,942	0,022	1	**0,007**	0,902	0,984
	GAD-7	0,977	0,028	1	0,416	0,924	1,033
	IIQ_Engulfment	1,581	0,150	1	**0,002**	1,177	2,123
	IIQ_Rejection	0,914	0,091	1	0,325	0,765	1,093
	IIQ_Acceptance	1,186	0,113	1	0,131	0,950	1,481
	IIQ_Enrichment	1,123	0,073	1	0,114	0,972	1,296
	HFERST_Rumination	1,049	0,079	1	0,542	0,899	1,225
	HFERST_Reappraisal	1,082	0,087	1	0,368	0,912	1,283
	HFERST_Acceptance	0,900	0,097	1	0,274	0,744	1,087
	HFERST_Problemsolving	1,117	0,103	1	0,285	0,912	1,367

HFERST_Suppression of expression	1,101	0,095	1	0,309	0,915	1,325
HFERST_Suppression of experience	1,237	0,109	1	0,052	0,998	1,532
HFERST_Avoidance	0,863	0,079	1	0,062	0,740	1,008
HFERST_Social sharing	1,057	0,066	1	0,405	0,928	1,202
Male	1,144	0,133	1	0,313	0,881	1,486
Female	0[a]		0			
Firm relationship	1,137	0,141	1	0,360	0,863	1,498
Single	0[a]		0			
Net income <=3000€	1,399	0,131	1	**0,011**	1,081	1,809
Net income >3000€	0[a]		0			
CHD simple	0,104	0,219	1	**<.001**	0,068	0,160
CHD moderate	0,422	0,146	1	**<.001**	0,317	0,561
CHD complex	0[a]		0			

Link Function: Logit. a. This parameter is set to zero because it is redundant. p values in bold indicate significant effects of the predictors.

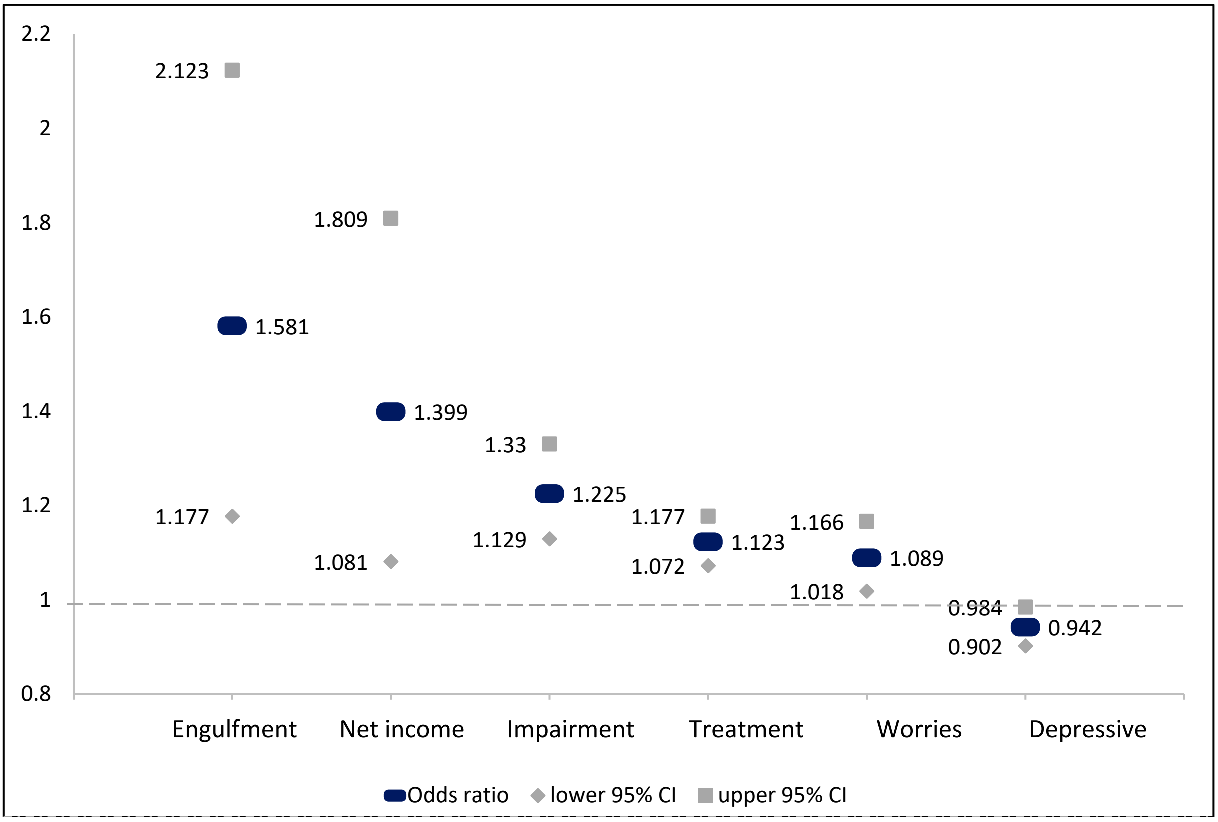

Figure S2. Odds ratios and 95%-CI intervals of significant independent variables in predicting the frequency of cardiological check-up intervals.
